# Biological Activities of Aerial Parts Extracts of* Euphorbia characias*

**DOI:** 10.1155/2016/1538703

**Published:** 2016-05-24

**Authors:** Maria Barbara Pisano, Sofia Cosentino, Silvia Viale, Delia Spanò, Angela Corona, Francesca Esposito, Enzo Tramontano, Paola Montoro, Carlo Ignazio Giovanni Tuberoso, Rosaria Medda, Francesca Pintus

**Affiliations:** ^1^Department of Public Health, Clinical and Molecular Medicine, University of Cagliari, Cittadella Universitaria, 09042 Monserrato, Italy; ^2^Department of Sciences of Life and Environment, University of Cagliari, Cittadella Universitaria, 09042 Monserrato, Italy; ^3^Department of Pharmacy, University of Salerno, 84084 Fisciano, Italy

## Abstract

The aim of the present study was to evaluate antioxidant, antimicrobial, anti-HIV, and cholinesterase inhibitory activities of aqueous and alcoholic extracts from leaves, stems, and flowers of* Euphorbia characias*. The extracts showed a high antioxidant activity and were a good source of total polyphenols and flavonoids. Ethanolic extracts from leaves and flowers displayed the highest inhibitory activity against acetylcholinesterase and butyrylcholinesterase, showing potential properties against Alzheimer's disease. Antimicrobial assay showed that leaves and flowers extracts were active against all Gram-positive bacteria tested. The ethanolic leaves extract appeared to have the strongest antibacterial activity against* Bacillus cereus* with MIC value of 312.5 *μ*g/mL followed by* Listeria monocytogenes* and* Staphylococcus aureus* that also exhibited good sensitivity with MIC values of 1250 *μ*g/mL. Moreover, all the extracts possessed anti-HIV activity. The ethanolic flower extract was the most potent inhibitor of HIV-1 RT DNA polymerase RNA-dependent and Ribonuclease H with IC_50_ values of 0.26 and 0.33 *μ*g/mL, respectively. The LC-DAD metabolic profile showed that ethanolic leaves extract contains high levels of quercetin derivatives. This study suggests that* Euphorbia characias* extracts represent a good source of natural bioactive compounds which could be useful for pharmaceutical application as well as in food system for the prevention of the growth of food-borne bacteria and to extend the shelf-life of processed foods.

## 1. Introduction

There has been a remarkable increment in scientific articles dealing with research of antioxidant molecules because of their protective action from the damage induced by oxidative stress. It causes serious cell and tissue damage leading it to be the major cause of the pathogenesis of several disease processes like cancer, diabetes, aging, and cardiovascular and neurodegenerative diseases. Plant materials represent a great source of antioxidant and bioactive compounds which are different in their composition and physical and chemical properties [1]. Phenolics are broadly distributed in the plant kingdom and are the most abundant secondary metabolites of plants. The identification and development of phenolic compounds or extracts from different plants has become a major area of health- and medical-related research. Among these compounds, flavonoids have been especially highlighted because they have been shown to have protective roles against many human diseases, due to their antioxidant capacity, which depends mainly on the number and position of hydroxyl groups within their structure, and their anti-inflammatory, anticancer, and antiviral activities [2]. Their activity as inhibitors of cholinesterase has also been demonstrated and correlated with their structure and could be useful for treatment of Alzheimer's disease (AD) [3]. AD results from a deficit of cholinergic functions in the brain. Hence, one of the most promising approaches for treating this disease is to restore the acetylcholine level by inhibiting cholinesterase activity. Acetylcholinesterase (AChE) and butyrylcholinesterase (BChE) are two cholinesterase enzymes that metabolize acetylcholine but differ in substrate specificity, enzyme kinetics, and activity in different brain regions. In a healthy brain, AChE is the most responsible enzyme in regulating acetylcholine levels but, in patients with AD, AChE activity gradually decreases with the concomitant increase of BChE. Thus, both AChE and BChE are legitimate therapeutic targets for treatment of cholinergic deficit characteristic of AD. Effective therapeutic options for AD are limited up to now; thus there is a demand for new natural drugs without side effects.

Moreover, the demand for bioactive compounds from natural sources as an alternative to synthetic molecules is continuously increasing also because of the emerging problem of the multidrug resistance of microorganisms related to antibiotics and their extensive use. Thus, several studies related to plant antimicrobials have demonstrated their efficacy towards a large number of pathogens and food-borne agents causing disease [4–6] as well as viral infections [7–10]. Although many plant-derived compounds are currently being used for the treatment of infectious diseases and for the preservation and extension of the shelf-life of foods, several medicinal and aromatic plants present worldwide remain still unexplored.

Euphorbiaceae is a large flowering plant family (300 genera and 8,000 species) widely distributed all around the world and composed of all sorts of plants (large woody trees, climbing lianas, or simple weeds) with a wide variety of chemical substances, many of them with a medicinal application. Some extracts from Euphorbiaceae plants have been characterized and patented as modern drugs [11]. Among Euphorbiaceae, the species* Euphorbia characias*, a nonsucculent shrub commonly occurring in vast areas of the Mediterranean region, has been analyzed and several biological active compounds were identified [12]. The plant latex has been the object of several researches and its screening has revealed the presence of natural rubber and numerous enzymes some of which interact in a common metabolism [13–22]. On the other hand, only little attention has been paid to the other parts of the plant [23–25].

Thus, the objective of this research was to evaluate antioxidant, antimicrobial, and anticholinesterase properties of the aqueous and ethanolic extracts of leaves, stems, and flowers from* E. characias* in order to find a novel potential source of bioactive molecules. Moreover, the antiviral efficacy of* E. characias* extracts was also evaluated on the human immunodeficiency virus type 1 (HIV-1) reverse transcriptase- (RT-) associated RNA-dependent DNA polymerase (RDDP) and Ribonuclease H (RNase H) activities. Both RT-associated functions are essential for viral replication and are validated drug targets for which new drugs are still needed [26, 27].

## 2. Materials and Methods

All chemicals were obtained as pure commercial products and used without further purification. Acetylcholinesterase (AChE) from* Electrophorus electricus*, acetylthiocholine iodide, 2,2′-azinobis-(3-ethylbenzothiazoline-6-sulfonic acid) (ABTS), aluminum nitrate, butyrylcholinesterase (BChE) from* equine serum*,* S*-butyrylthiocholine chloride, 3-*O*-caffeoylquinic acid (chlorogenic acid), catechin, 2,2-diphenyl-1-picrylhydrazyl radical (DPPH^•^), 5,5′-dithiobis(2-nitrobenzoic acid) (DTNB), ellagic acid, Folin-Ciocalteu phenol reagent, ferric chloride, galantamine hydrobromide, gallic acid, 4-hydroxybenzyl alcohol, 6-hydroxy-2,5,7,8-tetramethylchroman-2-carboxylic acid (Trolox), myricetin, 2,4,6-tris(2-pyridyl)-1,3,5-triazine (TPTZ), and quercetin were purchased from Sigma Aldrich (Milan, Italy). LC-MS grade acetonitrile and formic acid were purchased from Merck (Darmstadt, Germany). Standards of myricetin-3-*O*-glucoside, quercetin-3-*O*-glucoside, and acacetin were purchased from Extrasynthese (Genay, France). HPLC grade water (18 MΩ·cm) was prepared by using a Millipore (Bedford, MA, USA) Milli-Q purification system.

Spectrophotometric determinations were obtained with an Ultrospec 2100 spectrophotometer (Biochrom Ltd., Cambridge, England) using cells with a 1 cm path length.

### 2.1. Plant Material

**Figure 1:**
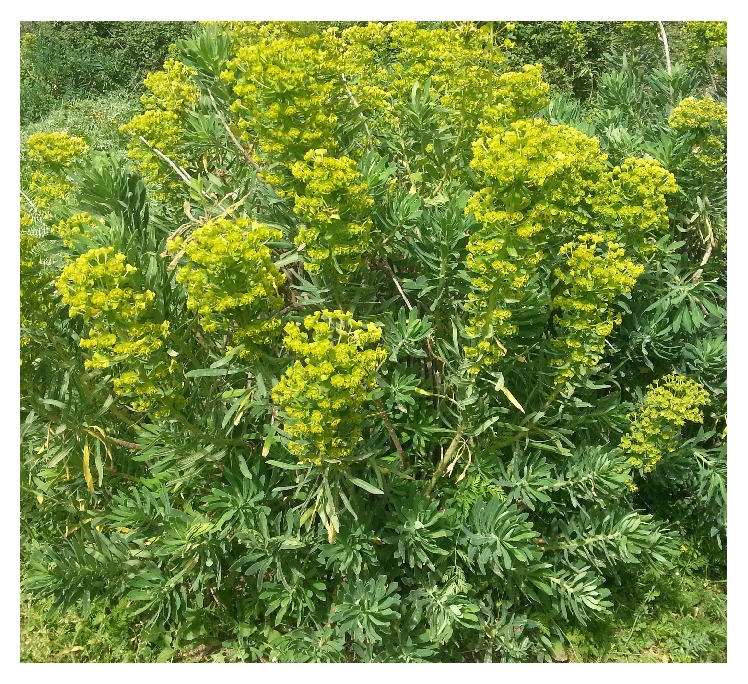
The Mediterranean shrub* Euphorbia characias* subsp.* characias*.

*E. characias* subsp.* characias* (Figure 1) was identified by Professor Annalena Cogoni and a voucher specimen has been deposited in the Department of Sciences of Life and Environment, University of Cagliari, Italy (number 1216/16 Herbarium CAG). The different parts of* E. characias* were collected from February to June, in southern Sardinia (Dolianova, CA, Italy). The GPS coordinates were 39°24′19.0′′N and 9°12′57.6′′E.

Leaves, stems, and flowers were immediately frozen at −80°C and then lyophilized in intact condition. The lyophilized plant materials (1 g) were reduced in powder and 10 mL of water (aqueous extract) or ethanol (ethanol extract) was added to the dried samples. The extraction was carried out in the dark at room temperature for 24 h under continuous stirring. Ethanol extracts were diluted 10-fold with water in order to freeze and then lyophilize the samples [24]. Before use, 1 mg of dried powders was dissolved in water or 10% ethanol (1 mL), for aqueous and ethanol extracts, respectively. For antimicrobial and antiviral activity dried powders were dissolved in DMSO (100%) as solvent. The yield (%, w/w) from all the dried extracts was calculated as follows: yield  (%) = (*A*1 × 100)/*A*2, where *A*1 is the weight of the dried extract (after lyophilization) and *A*2 is the weight of the plant powder.

### 2.2. Antioxidant Assays

In every extract total free radical scavenging molecules were determined by ABTS^•+^ and DPPH^•^ methods using Trolox as antioxidant standard, as previously reported [28, 29]. For both free radical methods, antioxidant activity was expressed as Trolox equivalent antioxidant capacity (TEAC; mmol/g dw). The FRAP (ferric reducing antioxidant power) assay was performed as previously described [30]. Quantitative analysis was performed according to the external standard method (FeSO_4_, 0.1–2 mmol/L, *r* = 0.9997) and results were expressed as mmol Fe^2+^/g dw.

### 2.3. Determination of the Total Polyphenols and Flavonoids

Total content of polyphenols and flavonoids in the extracts was determined as previously reported [29]. Polyphenol concentration was calculated using gallic acid as a referred standard and was expressed as mg of gallic acid (GAE) per 1 g of dry weight (dw). Flavonoid concentration was expressed as mg of quercetin equivalent (QE) per 1 g of dry extract.

### 2.4. Acetylcholinesterase and Butyrylcholinesterase Activity

Acetylcholinesterase from* Electrophorus electricus* and* equine serum* butyrylcholinesterase were used for the inhibitory assays. AChE activity was measured using Ellman's reagent according to the method previously reported [31]. Briefly, the reaction mixture contained 0.1 M phosphate buffer (pH 8.0), 1.5 mM 5,5′-dithiobis-2-nitrobenzoate (DTNB), acetylthiocholine iodide (1.5 mM), and extract at the desired concentrations or solvent alone (control) in a final volume of 1 mL. Finally, enzyme was added to the reaction mixture and the absorbance immediately monitored at 405 nm. For butyrylcholinesterase assay, the same procedure was followed except for the use of enzyme and substrate, which were BChE and* S*-butyrylthiocholine, respectively. Galantamine was used as the standard cholinesterase inhibitor. Results were expressed as IC_50_ values calculated as concentration of extracts that produces 50% cholinesterase activity inhibition.

### 2.5. Microbial Strains, Culture Conditions, and Antimicrobial Activity

*Staphylococcus aureus* ATCC 6538,* Bacillus cereus* ATCC 11178,* Listeria monocytogenes* ATCC 19115,* Escherichia coli* ATCC 35150 (serotype O157:H7),* Salmonella typhimurium* ATCC 14028,* Candida albicans* ATCC 10231,* Saccharomyces cerevisiae* ATCC 2601,* Aspergillus flavus* ATCC 46283 (aflatoxin producer), and* Penicillium chrysogenum* ATCC 10135 were obtained from the American Type Culture Collection (ATCC, Rockville, MD, USA) and used as indicators strains. All bacterial strains were stored on nutrient broth (NB, Microbiol, Cagliari, Italy) plus 20% (v/v) glycerol at −20°C except yeasts and molds strains which were maintained in potato dextrose broth (Microbiol) with 15% (v/v) glycerol. Before use, they were subcultured twice in appropriate medium.

Minimum inhibitory concentrations (MICs) and minimum bactericidal/fungicidal concentrations (MBCs/MFCs) of the* E. characias* extracts were determined by a broth microdilution method [5]. All tests were performed with NB for bacteria and RPMI 1640 (Sigma, Milan, Italy) buffered to pH 7.0 with morpholinepropanesulfonic acid (MOPS, Sigma) for yeasts and molds. The extracts were dissolved in DMSO (5% v/v). Serial doubling dilutions of each extract were performed in a 96-well microtiter plate ranging from 19.5 to 5000 *μ*g/mL. Overnight broth cultures were prepared in NB or RPMI and adjusted so that the final concentration in each well following inoculation was approximately 5.0 × 10^5^ cfu/mL. The concentration of each inoculum was confirmed using viable counts on Tryptic Soy Agar (TSA, Microbiol) plates for bacteria, Sabouraud Dextrose Agar (SDA, Microbiol) for yeasts, and potato dextrose agar (PDA, Microbiol) for molds. The controls included sterility of NB and RPMI broths, sterility of the extracts, control culture (inoculum), and control DMSO to check the effect of solvent on the growth of microorganisms. Furthermore, gentamicin, ketoconazole, and amphotericin B were used as positive controls for bacteria, yeasts, and molds, respectively.

The MICs and MBCs were determined after 24 h incubation of the plates at 37°C for bacteria and 30°C for fungi. Microbial growth was indicated by the presence of turbidity and a “pellet” on the well bottom. MICs were determined presumptively as the first well, in ascending order, which did not produce a pellet. To confirm MICs and to establish MBCs, 10 *μ*L of broth was removed from each well and inoculated on TSA, SDA, or PDA plates. After incubation under the conditions described above, the number of surviving microorganisms was determined. The MIC was the lowest concentration which resulted in a significant decrease in inoculum viability (>90%) while the MBC/MFC was the concentration where 99.9% or more of the initial inoculum was killed.

All tests were conducted in triplicate and with three replications, and the modal MIC and MFC values were selected.

### 2.6. HIV-1 RT-Associated Functions Biochemical Assays

HIV-1 RT gene subcloned into the p6HRT_prot plasmid was kindly provided by Stuart Le Grice (National Cancer Institute, Frederick, USA). Protein expression and purification was performed in* E. coli* M15 strain as described [32]. The HIV-1 RT-associated RDDP and RNase H activity were measured as previously described [33, 34].

### 2.7. LC Detection and Quantitative Analysis of Phenolic Compounds

#### 2.7.1. (HR) LC-ESI-Orbitrap-MS and (HR) LC-ESI-Orbitrap-MS/MS

The electrospray ionisation (ESI) source of a Thermo Scientific LTQ-Orbitrap XL (Thermo Scientific, Germany) mass spectrometer was tuned in negative ion mode with a standard solution of kaempferol-3-*O*-glucoside (1 *μ*g/mL) infused at a flow rate of 5 *μ*L/min with a syringe pump. In the FT experiment, resolution of the Orbitrap mass analyzer was set at 30000. The mass spectrometric spectra were acquired by full range acquisition covering *m*/*z* 120–1200 in LC-MS. The data recorded were processed with Xcalibur 2.0 software (Thermo Fisher Scientific). Initial calibration of the instrument was performed using the standard LTQ calibration mixture with caffeine and the peptide MRFA, dissolved in 50 : 50 (v/v) water/acetonitrile solution. LC/ESI/LIT-Orbitrap-MS was performed using a Finnigan Surveyor HPLC (Thermo Finnigan, San Jose, CA, USA) equipped with a Waters (Milford, MA, USA) Xselect CSH C18 3.5 *μ*m column (150 mm × 2.1 mm i.d.) and coupled to a hybrid Linear Ion Trap- (LIT-) Orbitrap mass spectrometer (Thermo Scientific). Linear gradient elution with a mobile phase comprising water acidified with 0.1% formic acid (solvent A) and acetonitrile acidified with 0.1% formic acid (solvent B) starting from 95% A was converted in 65% A in 45 min, from 65% to 0% (A) in 1 min, remaining 0% A for 4 minutes, and then from 0% to 95% (A) followed by 10 min of maintenance. The mobile phase was supplied at a flow rate of 200 *μ*L/min keeping the column at room temperature, and the effluent was injected directly into the ESI source. The mass spectrometer was operated in negative ion mode. ESI source parameters were as follows: capillary voltage −12 V; tube lens voltage −121.47 V; capillary temperature 280°C; sheath and auxiliary gas flow (N_2_) 30 and 5; sweep gas 0; and spray voltage 5 V. MS spectra were acquired by full range acquisition covering *m*/*z* 120–1600. LC-ESI-LIT-MS/MS data were obtained by applying a data dependent scan experiment, by directing to fragmentation the two highest peaks obtained in LC-ESI-Orbitrap-MS trace. Each parent ion was submitted to fragmentation with energy of 30% to produce an MS/MS spectrum in the MS range specific relative to its mass. 1 mg of dried extract obtained from ethanolic extract from* E. characias* leaves was dissolved in 10 mL of a mixture of water in acetonitrile and 10 *μ*L was injected in the LC-MS system.

#### 2.7.2. LC-DAD

Detection and quantitative analysis of phenolic compounds were carried out using an HPLC-DAD method [30]. Chromatograms and spectra were elaborated with a ChromQuest V. 2.51 data system (ThermoQuest, Rodano, Milan, Italy). Flavonols were detected and quantified at 360 nm and all the other compounds at 280 nm. Stock solutions were prepared at 1 mg/mL dissolving pure standards in methanol: water (50 : 50, v/v). The calibration curves for each compound were calculated by regression analysis, by plotting the peak area obtained after standards injection (3 replicates at each concentration) against the known standard concentrations. The stock solutions were diluted with methanol in order to obtain work solutions and the correlation values were 0.9992–0.9998.* E. characias* leaves extract was dissolved and injected in the LC-DAD system with the same condition of the LC-MS analysis.

### 2.8. Statistical Analysis

Data are reported as mean ± standard deviation of three independent experiments. The data were analyzed using one-way analysis of variance (one-way ANOVA) and Tukey's posttest. Statistical analysis was performed with GraphPad Prism 7 software (GraphPad Software, San Diego, California, USA). A difference was considered statistically significant at *p* < 0.05.

## 3. Results and Discussion

### 3.1. Antioxidant Activity, Polyphenol and Flavonoids Content, and Cholinesterase Activity Inhibition

Total free radical scavenging capacities determined with ABTS and DPPH assays are reported in Table 1. Comparable TEAC values of each extract were detected using the two methods. Leaves extracts exhibited significantly higher free radical scavenging activity (*p* < 0.05) than other extracts, with ethanolic extract showing the highest activity. Antioxidant activity was also examined with FRAP assay, confirming that ethanolic extract of leaves possessed the significantly highest antioxidant activity (*p* < 0.05), followed by flower ethanolic extract.

**Figure 2:**
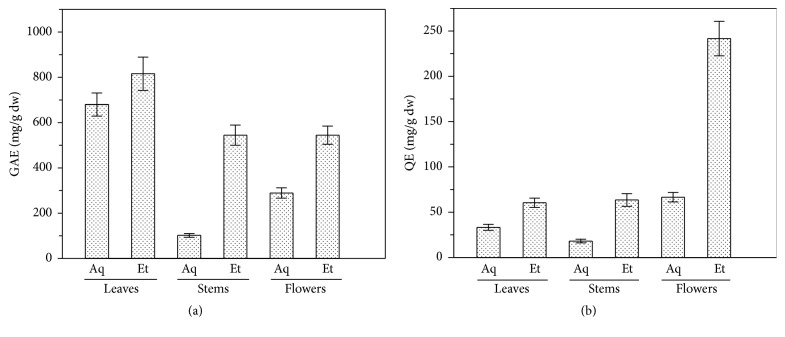
Polyphenol and flavonoid content in aqueous (Aq) and ethanolic (Et) leaves, stems, and flowers extracts from* E. characias*. (a) Polyphenol amount is expressed as mg of gallic acid equivalent (GAE) per g of dry weight (dw); (b) the amount of flavonoids is expressed as mg of quercetin equivalent (QE) per g of dry weight (dw). All data are expressed as mean of three measurements ± standard deviation.

**Figure 3:**
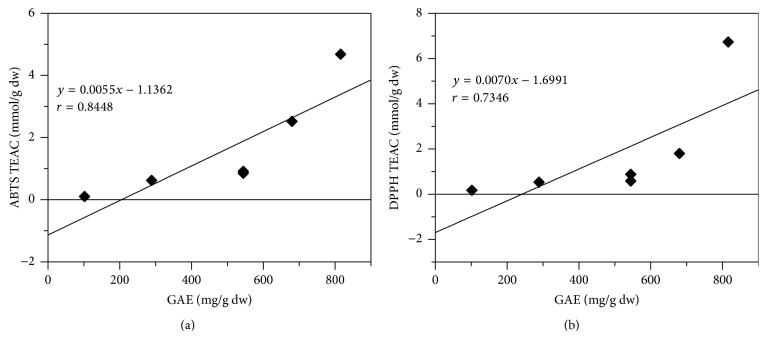
Correlation between phenolic content and antioxidant capacity of* E. characias* extracts. (a) ABTS assay; (b) DPPH assay. TEAC: Trolox equivalent antioxidant capacity; GAE: gallic acid equivalents.

The polyphenol and flavonoid content of the extracts, expressed as mg of gallic acid or quercetin equivalent, respectively, are reported in Figure 2. Total phenolic content varied widely among analyzed parts of the plant and the highest value was found in leaves in both aqueous and ethanolic extracts. In fact, the amount of phenolic compounds in leaves aqueous extract (680 mg GAE/g dw) was about 6- and 2.3-fold higher than the same extract from stems (102.1 mg GAE/g dw) and flowers (289.2 mg GAE/g dw), respectively (Figure 2(a)). Moreover, these results showed that ethanolic extract of leaves is rich in polyphenols content (815.7 mg GAE/g dw) with a value about 1.5-fold higher than the same extract from stems and flowers (about 544.6 mg GAE/g dw for each extract). Polyphenols have been reported to have antioxidant activity mainly based on their redox properties which have a key role in scavenging free radicals and chelating oxidant metal ions. High phenolic content in all* E. characias* extracts resulted in high TEAC values determined by ABTS or DPPH methods, which could indicate that phenolic compounds were capable of functioning as free radical scavengers. Good correlation was found between TEAC values for both ABTS and DPPH (*r* = 0.8448 and *r* = 0.7346, resp.) of the different plant extracts and their phenolic contents (Figure 3), confirming a strong relationship between antioxidant capacity and phenolic content.

Among phenolic compounds, flavonoids are ubiquitous plant compounds with an important role as attractants to pollinators, as sunscreens to protect against UV irradiation, and as antimicrobial and antiherbivory factors [37]. Content of flavonoids is shown in Figure 2(b). Flavonoid content of flowers ethanolic extract was about 2-fold higher (241.7 mg QE/g dw) than the correspondent extract from the other part of the plant (~62 mg QE/g dw). This extract also showed the highest ratio of total flavonoids (TFC) and phenolic content (TPC) being 0.44, indicating that flavonoids were almost 40% of the total phenolic content. However, aqueous extracts of both leaves and stems exhibited the lowest amounts of total flavonoids (33.2 mg and 18.1 mg QE/g dw, resp.), with aqueous extract from leaves showing the lowest TFC/TPC ratio of 0.05.

Compounds like flavonoids, alkaloids, terpenoids, and coumarins are known to have the capacity to inhibit cholinesterase types, key enzymes in the cholinergic nervous system [38]. This inhibitory activity is usually the first of a number of requirements for the development of medicines for treating some neurological disorders such as Alzheimer's disease.

Table 2 shows the acetylcholinesterase and butyrylcholinesterase inhibitory activities of the* E. characias* extracts, compared with those of standard inhibitor galantamine. A survey on IC_50_ values revealed that all the extracts exhibited acetylcholinesterase inhibitory activity while only few of them inhibited butyrylcholinesterase. This is not surprising because AChE and BChE enzymes display distinct substrate and inhibitor specificities. Most of the other IC_50_ values were about three orders of magnitude higher compared to the results obtained with galantamine. However, ethanolic leaves extract showed the significantly highest (*p* < 0.05) butyrylcholinesterase inhibition that is about 50 times less than the effect of galantamine. This is a good result since the standard inhibitor is a single molecule, whereas plant extracts are a mixture of numerous compounds and might contain only few active components.

### 3.2. Antimicrobial and Anti-HIV Activities

Tables 3(a) and 3(b) report the antagonistic activity of* E. characias* extracts against a panel of microorganisms including Gram-negative, Gram-positive, and spore-forming bacteria, yeast, and mold species. As can be observed, the leaves extracts appeared to have the strongest antibacterial activity followed by flowers and stems extracts. Gram-negative bacteria, yeasts, and molds were the least sensitive, being resistant to all extracts at the maximum concentration tested (5000 *μ*g/mL). Leaves extracts exhibited antibacterial activity towards all Gram-positive bacteria tested.* B. cereus* ATCC 11778 was the most susceptible strain being totally inhibited by the ethanolic leaves extract at the concentration of 312.5 *μ*g/mL (MIC equivalent to MBC).* L. monocytogenes* ATCC 19115 and* S. aureus* ATCC 6538 also exhibited a good sensitivity to the ethanolic leaves extract as they both showed MIC values of 1250 *μ*g/mL. Higher MIC values were observed for the aqueous leaves extracts towards these bacterial strains. As regards the flowers extracts, all Gram-positive strains tested were moderately inhibited by both aqueous and ethanolic extracts with MIC values equal to or greater than 2500 *μ*g/mL with the exception of* B. cereus* strain which was more sensitive to the ethanolic extract (MIC 1250 *μ*g/mL). Stems extracts showed the lowest activity, with ethanolic extract slightly inhibiting the abovementioned bacterial strains (MIC 5000 *μ*g/mL). Our findings are in agreement with those reported by Lin et al. for* Euphorbia macrorrhiza* species [39], who observed inhibitory activity against* S. aureus* but no effect towards* E. coli* and* C. albicans* strains tested. In contrast, Perumal et al. [40] observed antimicrobial effect for* Euphorbia hirta* ethanolic extracts towards both Gram-positive and Gram-negative bacteria with MIC values lower than those observed in this work. On the other hand, the antagonistic effect of plants extracts even within the same species is variable and depends on several factors such as the concentration of active components due to different tissue composition, variation in the extraction protocol and technique used to detect antimicrobial activity, and resistance of the test microorganisms [41–43].

Finally,* E. characias* extracts were also tested for their ability to inhibit the HIV-1 RT-associated RDDP and RNase H functions using the known nonnucleoside RT inhibitor efavirenz [27] and the diketo acid derivative RDS1759 [33] as control for RDDP and RNase H inhibition, respectively. Interestingly, all extracts were able to inhibit both RT functions (Table 4). In all cases, ethanolic extracts were more active than aqueous extracts and the flower extracts were the most potent on both enzyme activities.

### 3.3. LC-ESI-Orbitrap-MS, LC-ESI-Orbitrap-MS/MS, and LC-DAD Analysis of* E. characias* Ethanolic Leaves Extract

**Figure 4:**
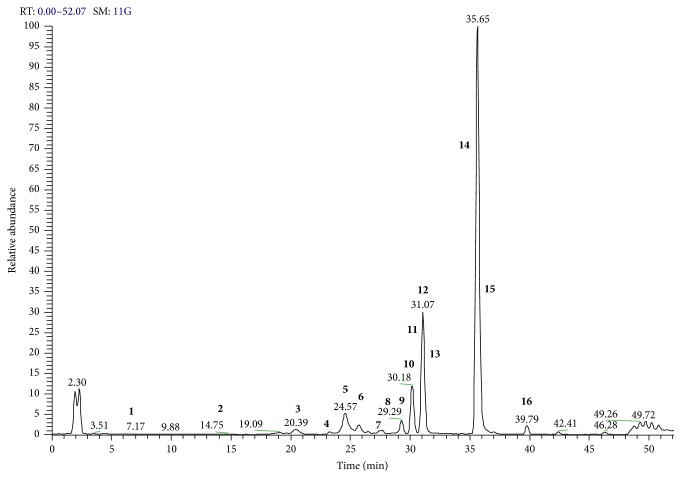
Identification of polyphenolic compounds in* E. characias* leaves using LC-ESI-Orbitrap-MS/MS in negative ion mode. Chromatographic conditions are described in the text. A list of compounds is reported in Table 5.

Considering all the antioxidants and biological activities of the extracts, ethanolic extract from leaves appeared to be the most active extract. Thus, this extract was selected for further study by liquid chromatography (LC). It was analyzed by an analytical method developed in LC-ESI-Orbitrap-MS and LC-ESI-Orbitrap-MS/MS, in negative ion mode. The negative LC-MS profile highlighted the presence of a large group of compounds corresponding to the deprotonated molecular ions of different flavonoids and ellagitannin derivatives (Figure 4). Individual components were identified by comparison of their *m*/*z* values in the Total Ion Current (TIC) profile with those of the selected compounds described in literature (Table 5). Additional LC-ESI-Orbitrap-MS/MS experiments were carried out in order to select and submit these ions to fragmentation experiments using the parameters previously chosen by ESI-MS and ESI-MS/MS direct infusion experiments. By matching experimental MS/MS spectra with those reported in literature and/or with those reported in a public repository of mass spectral data called MassBank [35], compounds** 1**–**16** were identified, with the exception of compounds** 3**,** 5**, and** 8** (unknown compounds).

Table 5 reports identification of compounds, based on high resolution mass spectrometric data, chemical formula derived by accurate mass maturation, retention times, MS/MS results, and references used for identification. Compounds** 1** and** 2** were identified by the diagnostic [M − H]^−^ ion shown in HR ESI-MS analysis, compared with standards and literature, and from the MS/MS data obtained working in LC-ESI-Orbitrap-MS/MS in Product Ion Scan in negative ion mode, and the compounds were identified by MassBank as gallic acid and catechin and confirmed by standard analysis. Compounds** 7**,** 9**,** 10**,** 11**, and** 15** were identified by the diagnostic [M − H]^−^ ions shown in HR ESI-MS analysis, their fragmentation profiles obtained in LC-ESI-(Orbitrap)-MS/MS in Product Ion Scan in negative ion mode, compared with literature data, and resulting compounds previously reported in* E. characias* leaves [25]. They are derivatives of quercetin. Compounds** 4** and** 6** were tentatively identified by the diagnostic [M − H]^−^ ions shown in HR ESI-MS analysis, their fragmentation profiles obtained in LC-ESI-Orbitrap-MS/MS in Product Ion Scan in negative ion mode, compared with MassBank data. They were proposed as myricetin derivatives. The identity of compound** 12** was hypothesized from the MSMS data obtained working in LC-ESI-Orbitrap-MS/MS in Product Ion Scan in negative ion mode, and the compound was tentatively identified by MassBank, as dicaffeoylquinic acid. Compounds** 13** and** 14** were tentatively identified by the diagnostic [M − H]^−^ ion shown in HR ESI-MS analysis and from the MS/MS data obtained working in LC-ESI-Orbitrap-MS/MS in Product Ion Scan in negative ion mode, compared with literature. These compounds were not described in* E. characias*, but in another plant of the same genus,* Euphorbia pekinensis* [36].

From a quantitative point of view, the most significant polyphenolic compounds were flavonoids, mainly quercetin derivatives (Table 5). Quercetin-3-(2-*O*-acetyl)-arabinoside (**15**) and quercetin-3-*O*-rhamnoside (**11**) were the most abundant compounds (39.99 ± 2.52 and 31.88 ± 2.75 g/L, resp.), followed by quercetin-3-*O*-arabinoside (**10**) and quercetin-3-*O*-xyloside (**9**). These findings are in agreement with the results of previous investigations on the aerial part of* E. characias* [25]. For the first time other compounds such as gallic acid (**1**), catechin (**2**), myricetin derivatives, and ellagic acid derivatives were quantified in* E. characias* leaves extracts. Some of these compounds such as gallic acid, catechin, and quercetin-3-*O*-rhamnoside were isolated from other plant sources and displayed cholinesterase inhibitory activity [44–46]. Several compounds are derivatives of myricetin (**4** and** 6**) and quercetin (**7**,** 9**–**11,** and** 15**), two flavonoids which showed anti-HIV-1 activity [8, 47]. Finally, previous studies indicated that quercetin derivatives possess antimicrobial properties and the mechanisms of these compounds were attributed to their ability to form complexes with extracellular and soluble proteins and bacterial cell walls [48]. This might explain the lack of activity or the minor susceptibility of the Gram-negative bacteria and the greater inhibition of* E. characias *extract against Gram-positive bacteria. In fact, Gram-negative bacteria possess an effective permeability barrier represented by the outer lipidic membrane which could restrict the penetration of plant extracts. The next step will be the isolation of pure compounds of the extract and the evaluation and characterization of their biological activities in order to find new bioactive compounds from a natural source.

## 4. Conclusions

The results of this study indicate that* E. characias* aerial parts are a good source of antioxidant, antimicrobial, antiviral, and anti-ChE compounds. Leaves extracts exhibited the strongest antioxidant activities and the highest amount of polyphenols and cholinesterase inhibitors molecules, while flowers extracts seemed to be the best sources of flavonoids and exerted the best antiviral activity. Extracts also exhibited acetyl- and butyrylcholinesterase inhibition and these are promising results since ChE inhibitors represent the standard therapeutic approach to the treatment of Alzheimer's disease.

Analyzing all results, extraction with ethanol was more efficient than use of plain water. In fact ethanol extracts were found to provide the highest antioxidant and anti-ChE activity and phenolic and flavonoids contents compared with the correspondent aqueous extract. The same result was found for antimicrobial assays since ethanol extracts were found to be more effective towards the food-borne pathogens* L. monocytogenes*,* S. aureus*, and* B. cereus*. HPLC-DAD and LC-ESI-MS chromatogram revealed that leaves ethanolic extract showed the presence of several phenolic and flavonoid compounds known for their antioxidant activity and that could be responsible for the biological activity of the extract. Further studies will be performed for the isolation and characterization of single active compounds, which could be used for pharmaceutical application as well as in food system for the prevention of the growth of food-borne bacteria.

## Figures and Tables

**Table 1:** Yield and antioxidant and antiradical properties of *E. characias* extracts. DPPH and ABTS values are expressed as mmol TEAC/g dw; FRAP value is expressed as mmol Fe^2+^/g dw.

Extracts		Yield (w/w%)	ABTS	DPPH	FRAP
Leaves	Aqueous	20.4 ± 1.5	2.52 ± 0.18^b^	1.8 ± 0.21^b^	2.70 ± 0.17^c^
	Ethanolic	24.9 ± 2.6	4.68 ± 0.49^a^	6.73 ± 0.70^a^	4.57 ± 0.12^a^

Stems	Aqueous	12.3 ± 1.4	0.10 ± 0.01^d^	0.17 ± 0.02^c^	0.89 ± 0.04^f^
	Ethanolic	15.2 ± 1.8	0.85 ± 0.10^c^	0.88 ± 0.09^c^	1.36 ± 0.07^e^

Flowers	Aqueous	17.6 ± 2.3	0.62 ± 0.05^cd^	0.53 ± 0.05^c^	1.95 ± 0.02^d^
	Ethanolic	20.6 ± 1.9	0.91 ± 0.10^c^	0.58 ± 0.05^c^	3.49 ± 0.09^b^

Results are expressed as mean ± standard deviation of three independent experiments. Means followed by distinct letters in the same column are significantly different (*p* < 0.05).

**Table 2:** Inhibition of AChE and BChE by *E. characias* extracts.

Extracts		IC_50_ values (mg/mL)	
		AChE	BChE
Leaves	Aqueous	4.2 ± 0.25^c^	—
	Ethanolic	0.6 ± 0.056^d^	0.39 ± 0.04^c^

Stems	Aqueous	6.9 ± 0.71^a^	—
	Ethanolic	5.8 ± 0.43^b^	—

Flowers	Aqueous	5.25 ± 0.35^b^	4.2 ± 0.39^a^
	Ethanolic	0.6 ± 0.045^d^	1.22 ± 0.08^b^

Galantamine		0.27 ± 0.07 *μ*g/mL	8.12 ± 0.61 *μ*g/mL

Results are expressed as mean ± standard deviation of three independent experiments. Means followed by distinct letters in the same column are significantly different (*p* < 0.05).

**(a):** 

Target microorganisms	Leaves		Stems		Flowers	
	MIC	MBC/MFC	MIC	MBC/MFC	MIC	MBC/MFC
*E. coli*	>5000	—	>5000	—	>5000	—
*S. typhimurium *	>5000	—	>5000	—	>5000	—
*S. aureus*	5000	>5000	>5000	—	5000	>5000
*B. cereus*	1250	1250	5000	>5000	2500	5000
*L. monocytogenes*	2500	2500	>5000	—	2500	>5000
*C. albicans*	>5000	—	>5000	—	>5000	—
*S. cerevisiae*	>5000	—	>5000	—	>5000	—
*A. flavus*	>5000	>5000	>5000	—	>5000	>5000
*P. chrysogenum*	>5000	>5000	>5000	—	>5000	>5000

**(b):** 

Target microorganisms	Leaves		Stems		Flowers	
	MIC	MBC/MFC	MIC	MBC/MFC	MIC	MBC/MFC
*E. coli*	>5000	—	>5000	—	>5000	—
*S. typhimurium *	>5000	—	>5000	—	>5000	—
*S. aureus*	1250	2500	5000	>5000	5000	>5000
*B. cereus*	312.5	312.5	5000	>5000	1250	2500
*L. monocytogenes*	1250	1250	5000	>5000	5000	>5000
*C. albicans*	>5000	—	>5000	—	>5000	—
*S. cerevisiae*	>5000	—	>5000	—	>5000	—
*A. flavus*	>5000	—	>5000	—	>5000	>5000
*P. chrysogenum*	>5000	—	>5000	—	>5000	>5000

Positive controls: ampicillin MICs (*S. aureus* ATCC 6538: 2.5 *μ*g/mL, *B. cereus* ATCC 11778: 10 *μ*g/mL, and *L. monocytogenes* ATCC 19115: 2.5 *μ*g/mL); gentamicin MICs (*E. coli* ATCC 35150: 10 *μ*g/mL, *S. typhimurium* ATCC 14028: 10 *μ*g/mL); ketoconazole MICs (*C. albicans* ATCC 10231: 2.5 *μ*g/mL, *S. cerevisiae* ATCC 2601: 2.5 *μ*g/mL); amphotericin B MICs (*A. flavus* ATCC 46283 and *P. chrysogenum* ATCC 10135: 5 *μ*g/mL).

**Table 4:** Effects of *E. characias* extracts on HIV-1 RT-associated functions.

Extracts		IC_50_ (*μ*g/mL)^*∗*^	
		HIV-1	HIV-1
		RDDP	RNase H
Leaves	Aqueous	0.785 ± 0.003^c^	1.95 ± 1.03^a,b^
	Ethanolic	0.75 ± 0.028^c^	0.685 ± 0.155^b,c^

Stems	Aqueous	6.87 ± 0.81^a^	2.235 ± 0.245^a^
	Ethanolic	3.05 ± 0.2^b^	1.615 ± 0.035^a,b,c^

Flowers	Aqueous	1.03 ± 0.0^c^	1.51 ± 0.53^a,b,c^
	Ethanolic	0.26 ± 0.08^c^	0.33 ± 0.1^c^

Efavirenz		0.0016 ± 0.0003^*∗∗*^	ND^*∗∗∗*^

RDS1759		ND	7.1 ± 0.5^*∗∗*^

^*∗*^Extracts concentration required to inhibit HIV-1 RT-associated functions by 50%.

^*∗∗*^Values expressed in *μ*M concentration.

^*∗∗∗*^Not done.

Means followed by distinct letters in the same column are significantly different (*p* < 0.05).

**Table 5:** Identification of polyphenolic compounds in *E. characias* leaves ethanolic extract using LC-ESI-Orbitrap-MS/MS in negative ion mode and quantification by LC-DAD.

	Putative identification	RT (min)	g/L (mean ± SD)	MW	[M − H]^−^	Molecular formula	MS/MS	References
**1**	Gallic acid^a^	7.17	0.94 ± 0.23	170.0215	169.1195	C_7_H_6_O_5_	125.02	[35]
**2**	Catechin^a^	14.75	0.65 ± 0.04	290.2680	289.0715	C_15_H_13_O_6_	245.05 205.04 125.02	[35]
**3**	Unknown	20.39	NQ		951.0734		932.70	—
**4**	Myricetin-hexose^b^	23.16	0.02 ± 0.00	480.0904	479.0824	C_21_H_19_O_13_	317.06	[35]
**5**	Unknown	24.56	NQ		960.789		913.01	—
**6**	Myricetin-deoxyhexose^b^	26.49	0.01 ± 0.00	464.0954	463.0873	C_21_H_19_O_12_	317.06	[35]
**7**	Quercetin-3-*O*-glucoside^a^	27.52	1.94 ± 0.09	464.0954	463.0873	C_21_H_19_O_12_	301.07	[25]
**8**	Unknown	28.97	NQ		469.0516	C_22_H_13_O_12_	393.07	—
**9**	Quercetin-3-*O*-xyloside^c^	29.20	2.25 ± 0.16	434.0849	433.0771	C_20_H_17_O_11_	301.07	[25]
**10**	Quercetin-3-*O*-arabinoside^c^	30.18	9.54 ± 0.36	434.0849	433.0771	C_20_H_17_O_11_	301.07	[25]
**11**	Quercetin-3-*O*-rhamnoside^a^	31.02	31.88 ± 2.75	448.1005	447.0924	C_21_H_19_O_11_	301.25	[25]
**12**	Di-*O*-caffeoylquinic acid^d^	31.07	0.02 ± 0.00	516.0962	515.0800	C_17_H_23_O_18_	353.10 191.02	[35]
**13**	3,3′-Dimethyl ellagic acid pentose^e^	31.53	0.01 ± 0.00	462.0798	461.0877	C_22_H_19_O_12_	329.02	[36]
**14**	3,3′-Dimethyl ellagic acid deoxyhexose^e^	35.70	0.01 ± 0.00	476.0954	475.0877	C_22_H_19_O_12_	329.02	[36]
**15**	Quercetin-3-(2-*O*-acetyl)-arabinoside^a^	35.74	39.99 ± 2.52	476.0954	475.0877	C_22_H_19_O_12_	300.08	[25]
**16**	Acacetin glucuronide^f^	39.84	0.50 ± 0.02	460.1005	459.0923	C_22_H_19_O_11_	283.27	[35]

^a^Quantified using corresponding authentic standard; ^b^quantified as equivalent of myricetin-3-*O*-glucoside; ^c^quantified as equivalent of quercetin-3-*O*-glucoside; ^d^quantified as equivalent of chlorogenic acid; ^e^quantified as equivalent of ellagic acid; ^f^quantified as equivalent of acacetin; NQ: not quantified.

## Abbreviations

AChE: Acetylcholinesterase
AD: Alzheimer's disease
ABTS: 2,2′-Azinobis-(3-ethylbenzothiazoline-6-sulfonic acid)
BChE: Butyrylcholinesterase
DPPH^•^: 2,2-Diphenyl-1-picrylhydrazyl radical
FRAP: Ferric reducing antioxidant power
GAE: Gallic acid equivalent
MBCs/MFCs: Minimum bactericidal/fungicidal concentrations
MICs: Minimum inhibitory concentrations
QE: Quercetin equivalent
RDDP: RNA-dependent DNA polymerase.
